# An Efficient Key Generation for the Internet of Things Based Synchronized Quantization

**DOI:** 10.3390/s19122674

**Published:** 2019-06-13

**Authors:** Mike Yuliana, Wirawan, Suwadi

**Affiliations:** 1 Department of Electrical Engineering, Faculty of Electrical Technology, Institut Teknologi Sepuluh Nopember, Jalan Raya ITS, Keputih, Sukolilo, Surabaya 60111, Indonesia; wirawan@ee.its.ac.id (W.); suwadi@ee.its.ac.id (S.); 2 Department of Electrical Engineering, Politeknik Elektronika Negeri Surabaya (PENS), Jalan Raya ITS, Keputih, Sukolilo, Surabaya 60111, Indonesia

**Keywords:** Internet of Things, signal strength exchange system, synchronized quantization

## Abstract

One solution to ensure secrecy in the Internet of Things (IoT) is cryptography. However, classical cryptographic systems require high computational complexity that is not appropriate for IoT devices with restricted computing resources, energy, and memory. Physical layer security that utilizes channel characteristics is an often used solution because it is simpler and more efficient than classical cryptographic systems. In this paper, we propose a signal strength exchange (SSE) system as an efficient key generation system and a synchronized quantization (SQ) method as a part of the SSE system that synchronizes data blocks in the quantization phase. The SQ method eliminates the signal pre-processing phase by performing a multi-bit conversion directly from the channel characteristics of the measurement results. Synchronization is carried out between the two authorized nodes to ensure sameness of the produced keys so it can eliminate the error-correcting phase. The test results at the IoT devices equipped with IEEE 802.11 radio show that SSE system is more efficient in terms of computing time and communication overhead than existing systems.

## 1. Introduction

Today the Internet of Things (IoT) has become a part of our activities with many interconnected things that can be controlled by the internet [1,2,3,4,5]. This condition results in the emergence of new security threats that opens opportunities for third parties to access and obtain confidential information. Most of these things are connected using radio technology which creates communication become susceptible to tapping [6,7,8,9]. Many classic security services are realized in the upper Open System Interconnection (OSI) layers, for example, encryption schemes based on key distribution and computational complexity [10,11]. The scheme requires high computational complexity so it is not suitable for IoT devices that have limited computing resources, energy, and memory. 

Several studies have focused on security systems from devices with restricted resources and lightweight cryptographic to resolve this issue. One solution is to propose chaotic cryptosystem by optimizing and designing complementary metal-oxide-semiconductor (CMOS) that is connected with chaotic oscillators so that it can be developed in various communication security applications of IoT devices [12,13]. The other interesting solution of lightweight cryptographic is a key generation system that generates an encryption key by using the channel characteristics of the measurement results [14,15,16,17,18]. The system utilizes the principle of reciprocity from electromagnetic propagation which shows that the channel characteristics obtained by the sender and receiver will be the same if the measurement is carried out within coherence time. Some studies use the received signal strength (RSS) as one of the characteristics of radio channels [19,20,21,22,23]. These channel characteristics are the most available characteristics in wireless devices with various standards, i.e., Bluetooth, IEEE 802.15.4, and IEEE 802.11. 

The principle of channel reciprocity as the basis of key generation shows the similarity of the produced channel characteristics from both users. In reality, however, most wireless devices work by alternating measurements of channel characteristics. This condition results in a decrease similarity of channel characteristics due to non-simultaneous measurements and wireless devices noise. Several studies have attempted to improve the similarity of produced channel characteristics by appending the signal pre-processing phase after the measurement process [24,25,26,27,28,29]. The addition of this phase is proven to be able to raise the similarity of the channel characteristics as indicated by an increase in the correlation coefficient value. The higher the correlation coefficient value of the channel characteristics, the more likely it is to get the same key between the two users. The weakness of the research is that there is still a possibility of different key between the two users so the error-correcting phase is needed to create an equal key. The more the key bits to be corrected then the longer the computing time is needed to make corrections. This phase also increases the communication time between two users due to the exchange of parity bits. 

Some studies [30,31] seek to overcome this problem by modifying the existing signal pre-processing phase. The phase is modified by dividing the measurement data into several blocks of data. Each data block will be pre-processed using the existing signal pre-processing method. Another study [32] modified the signal pre-processing phase by combining the Polynomial Regression method with the modified Kalman Filter. The phase is also carried out in each block of measurement data. The results of the performed tests indicate that the use of modified signal pre-process phase is able to produce the same key bits without going through the error correcting phase. However, the signal pre-processing phase requires high computing time because it is conducted for each data block. The more the data amount is, then higher computing time is needed. The frequently parameters for determining the success of the built key generation system are the correlation coefficient, key discrepancy rate, key synchronized rate, and irregularity. The correlation coefficient is used to determine the increasing similarity of the produced channel characteristics. The key synchronized rate is used to specify the number of key bits produced during the duration of the key generation system communication. The duration time of key generation includes the measurement time of channel characteristics, the computation time of each phase and the communication/synchronization time between the two users. Communication/synchronization time obtained if there is an exchange of information between the two users. Phases that often require synchronization are error correcting and privacy amplification phases. The more information sent, the higher the synchronization time needed so the duration of the key generation will also be longer. The higher the duration of communication, the lower the key synchronized rate obtained. The key discrepancy rate is used to specify the number of key bits that are different from the two users. The irregularity is used to determine randomness of key bits generated using the National Institute of Standards and Technology (NIST) test. Some studies on the key generation system for IoT devices [6,33,34] also focus on using these parameters to determine the performance of the built key generation system. The parameters of performance in IoT devices shall be added with computing time and communication overhead that will show the efficiency of the system. The lower the computing time and communication overhead the more efficient the system is, so it is suitable to be implemented on devices with limited resources. 

In this paper, we propose an efficient key generation design without the signal pre-processing and error-correcting phase so it can reduce computing time and communication overhead. The details on the two contributions made in this paper will be abbreviated as follows. Firstly, we propose a new quantization method i.e., synchronized quantization (SQ) as a part of the signal strength exchange (SSE) system. The method utilizes the mean, standard deviation and a parameter as the standard deviation dividing parameter. The bits conversion results of the SQ method are then divided into several blocks, each containing 3 bits. The bits taken as key extraction are the three bits totaling 0 or 3. In this paper, we synchronize key bits between the two users by sending the wasted index of blocks. The synchronization system is able to produce the same key bits without going through the signal pre-processing and error-correcting phases. Compared to the previous system, the performance evaluations of the proposed system show faster computing time and lower communication overhead. Secondly, we validate the performance of the SSE system in two real indoor environment scenarios, i.e., unobstructed and obstacles scenarios. Performance validation is conducted by using several performance parameters, i.e., key synchronized rate (KSR), key discrepancy rate (KDIR), irregularity, computing time, and communication overhead.

The rest of this paper is arranged as follows. Section 2 describes in detail about the introduction of key generation models and principle. Section 3 provides an overview of existing key generation system. Section 4 explains in detail about the proposed key generation system, i.e., the SSE System. Section 5 discusses the implementation and performance evaluation of the SSE key generation system. Section 6 summarizes the performance of the built key generation system.

## 2. Key Generation Model and Principle

In this section, we explain the key generation model and principle. The key generation model is used to indicate the involved node/user and the type of attack from the attacker. The principle key generation explains the three principles used in key generation systems. 

### 2.1. Key Generation Model

**Figure 1:**
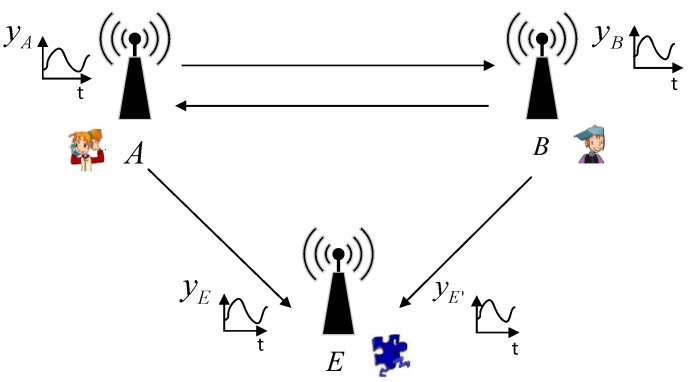
Key generation model.

There are three nodes involved in this paper Alice (A), Bob (B) and Eve (E) as seen in Figure 1. Authorized nodes are A and B, while an unauthorized node is E. A and B conduct the measurement process to get the channel characteristics $y_{A}$ dan $y_{B}$. Based on the principle of channel reciprocity, authorized nodes will get almost the same or identical channel characteristics measurement results $\left(y_{A}\approx y_{B}\right)$ if the measurement were performed within coherence time [35]. E as an unauthorized node listens to all the probing processes so it will get the channel characteristics from A and B such as $y_{E}$ and $y_{E^{\prime }}$. In this paper, there are three assumptions that will be used for the node E. The first assumption E can listen to all communications made by authorized nodes. In addition, the distance of E is also more than ½ wavelength from the authorized nodes so the received channel characteristics will not correlate with the produced channel characteristics from the authorized nodes $\left(y_{E}\neq y_{A},y_{E^{\prime }}\neq y_{B}\right)$. The second assumption E is a passive tapper, so it will not attack the communications made by authorized nodes. The third assumption E knows all the algorithms used by authorized nodes. 

### 2.2. Key Generation Principle

The three principles used in key generation systems include channel reciprocity, temporal variation, and spatial decorrelation [36]. The principle of channel reciprocity shows that channel characteristics are measured by authorized nodes and they will be the same if measured simultaneously. However, most wireless devices have limited capabilities so they are not able to take measurements simultaneously. This condition results in the nonidentical channel characteristics between the two authorized nodes. The value of the channel reciprocity of both authorized nodes can be measured by Equation (1). The principle of temporal variation can be obtained if there is a movement of the sender or recipient node and other things in the observation area. The movement of objects and nodes can affect the level of randomness of the produced channel characteristics. The higher the level of randomization, the more difficult it is for tappers to get the same key as the authorized nodes. The last principle the spatial decorrelation is very important to determine the security of the built key generation system. In this principle, the distance of the unauthorized node that is more than ½ wavelengths will get uncorrelated channel characteristics with the authorized nodes [37].

(1) $$r_{y_{A}y_{B}}=\frac{\sum_{i=1}^{n}\left(y_{A_{i}}-\mu_{A})(y_{B_{i}}-\mu_{B}\right)}{\sqrt{\sum_{i=1}^{n}{\left(y_{A_{i}}-\mu_{A}\right)}^{2}}\sqrt{\sum_{i=1}^{n}{\left(y_{B_{i}}-\mu_{B}\right)}^{2}}}$$

where $r_{y_{A}y_{B}}$ is the correlation coefficient of characteristics of the channel measurement results between the two authorized nodes, n states the number of channel characteristics, whereas $\mu_{A}$ and $\mu_{B}$ is the average of the channel characteristics measurement results of A and B. $\mu_{A}$ is obtained by $\mu_{A}=\frac{1}{n}\sum_{i=1}^{n}y_{A_{i}}$ and $\mu_{B}$ is obtained by $\mu_{B}=\frac{1}{n}\sum_{i=1}^{n}y_{B_{i}}$. 

## 3. Key Generation System Overview

**Figure 2:**
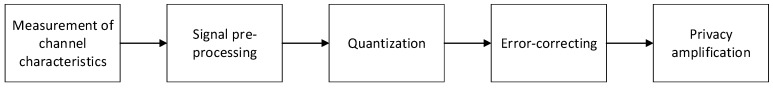
The Existing of the key generation system.

Some existing studies [24,25,26,27,28,29] use a key generation system consisting of five phases as shown in Figure 2. The first phase is to alternately measure channel characteristics between two authorized nodes. Non-simultaneous measurements can lead to high differences in the generated key bits. To overcome these problems, the signal pre-processing phase is carried out on measured channel characteristics using smoothing [24] and filtering [28]. The third phase is quantization which aims to change the channel characteristics of the signal pre-processing results in the form of a single bit [38] and multi-bit [39]. The fourth phase is the error-correcting that is used to correct the different bits due to nonsimultaneous measurements. The last phase is privacy amplification which aims to obscure the part of the leaked key to the tapper by increasing the randomness of the produced key. At this step, verification is also carried out which aims to ensure that the produced keys from the authorized nodes are the same. 

The other studies use a key generation system consisting of four phases [40,41,42]. These phases include the measurement of channel characteristics, quantization, error-correcting, and privacy amplification. The performance evaluation shows that direct quantization can result in high bit mismatches at the quantization phase. The addition of the signal pre-processing phase at [24,25,26,27,28,29] aims to reduce the incompatibility of the resulting bits. However, compared with [32], the studies that use five phases tend to be less efficient due to the addition of the signal pre-processing and error-correcting phases, which increases computing time. The higher the difference bit that occurs, the higher the produced computing time is because more data blocks must be corrected. The study [32] modified the signal pre-processing phase to increase the similarity of channel characteristics in each block of measurement data and eliminate error-correcting phase. However, the signal pre-processing phase requires high computing time because it is conducted for each data block. The more data the higher computing time needed. In this paper, we try to overcome this problem by proposing a simpler key generation system, i.e., signal strength exchange (SSE) system because it only consists of 3 phases. Elimination of signal pre-processing and error-correcting phases significantly reduced computing time so it is suitable for IoT devices with limited computing resources. 

## 4. SSE System 

**Figure 3:**
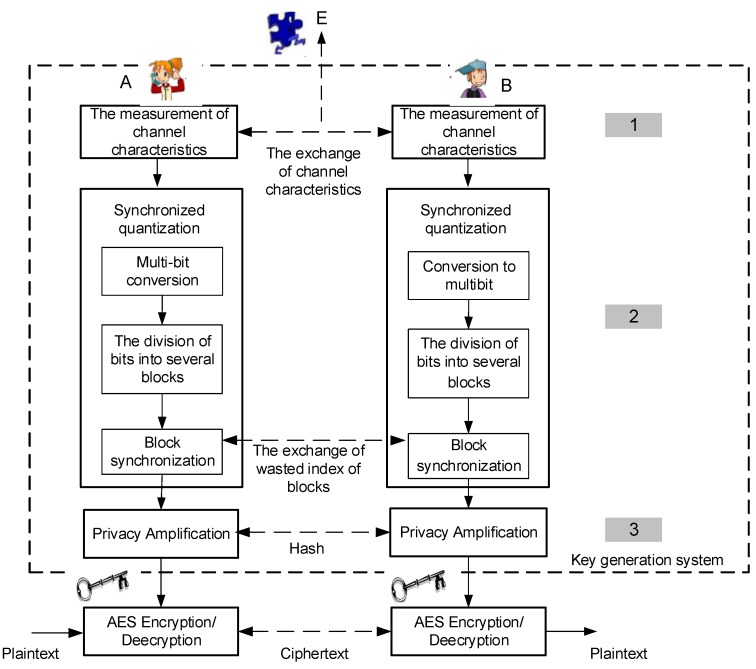
SSE key generation system.

We propose the SSE system as a key generation system that consists of three phases, i.e., measurement of channel characteristics, quantization, and privacy amplification as shown in Figure 3. The system is simpler when compared to existing systems [24,25,26,27,28,29,32,40,41,42], so it is expected to be able to reduce computing time, communication overhead and suitable for IoT devices with limited computing resources. We claim that our proposed key generation system is simpler in terms of the number of phases that must be passed. The more phases that must be passed will affect the produced computing time. The amount of communication/synchronization between two nodes will affect communication overhead and the produced communication/synchronization time. Table 1 shows the comparison of the phases that must be passed on the key generation. Overall, it can be said that SSE systems have fewer phases compared to existing systems. The measurement of channel characteristics was carried out in two different scenarios i.e., unobstructed and obstacles scenarios. In this paper, we use RSS as channel characteristics to be measured due to the most easily acquired on a variety of wireless devices. A is one of the authorized nodes selected as the initiator. The channel characteristics of measurement results will be converted into multi-bit using the quantization phase. As part of the SSE system, we also propose a new quantization method i.e., synchronized quantization (SQ) which synchronizes data blocks in the quantization phase. Synchronization is carried out between the two authorized nodes by exchanging wasted index of blocks to ensure that the produced key is absolutely the same so it can eliminate the error-correcting phase. Another advantage of the SQ method is the elimination of the signal pre-processing phase. This occurs because the SQ method is able to produce absolutely the same key bits directly from the channel characteristics of the measurement results. In the privacy amplification phase, we use the universal hash [43] to obfuscate the leaked key parts to the unauthorized node and SHA-1 [44] to ensure that the produced keys by the two authorized nodes are the same. 

**Figure 4:**
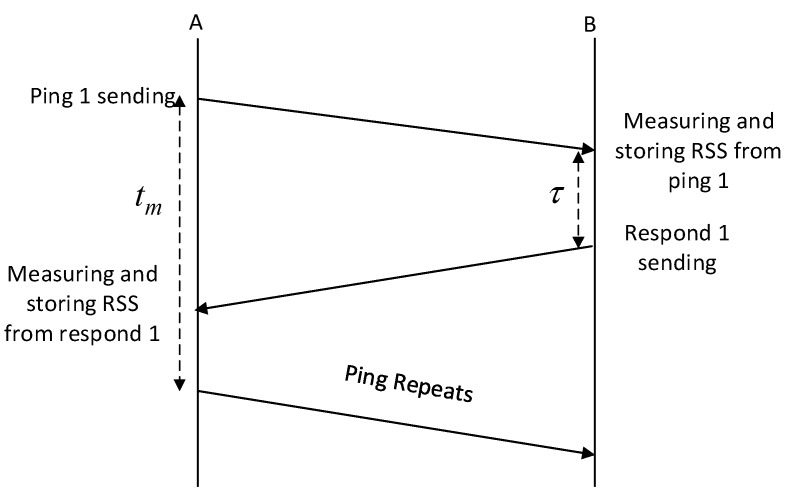
The mechanism of RSS channel characteristics measurement.

The first phase of the SSE system is the mechanism for measuring RSS channel characteristics $y_{u}$ between nodes as shown in Figure 4. Subscript u can be replaced with A for Alice, B for Bob, E for Eve (RSS channel characteristics from A), and $E^{\prime }$ for Eve (RSS channel characteristics from B). A sends pings to B with each time interval $t_{m}$. B measures and stores the RSS channel characteristics of the ping conducted by A before giving response with delay τ. In the same way, A also conducts measurement and stores the RSS channel characteristics of response conducted by B. To ensure the reciprocity of the produced channel characteristics τ must be made as small as possible so it is smaller than coherence time. From the measurement phase, A and B collect a number of RSS channel characteristics n as shown by Equations (2) and (3). E as an unauthorized node listens to all the measurement processes and collects a number of RSS channel characteristics from A and B as seen in Equations (4) and (5).

(2) $$y_{A}={\left[y_{A}(1),y_{A}(2),\ldots ,y_{A}(n)\right]}^{T}$$

(3) $$y_{B}={\left[y_{B}(1),y_{B}(2),\ldots ,y_{B}(n)\right]}^{T}$$

(4) $$y_{E}={\left[y_{E}(1),y_{E}(2),\ldots ,y_{E}(n)\right]}^{T}$$

(5) $$y_{E^{\prime }}={\left[y_{E^{\prime }}(1),y_{E^{\prime }}(2),\ldots ,y_{E^{\prime }}(n)\right]}^{T}$$

The second phase is to convert the RSS channel characteristics $y_{u}$ into multi-bit using the SQ method. This method utilizes three parameters, i.e., standard deviation $\sigma_{u}$, mean $\mu_{u}$ and a to determine the produced bits in each area. In this paper standard deviation and mean are calculated from the overall RSS channel characteristics, while *a* is used as dividers from the standard deviation. These parameters are used to determine the area of each channel characteristics. Bit conversion $Q_{u}$ from each area determined using Gray code as shown by Equation (6). All the RSS channel characteristics will be converted into multi-bit and no waste channel characteristics. The resulting multi-bit sequence $K_{u}$ is seen in Equation (7). 

(6) $$Q_{u}=\left\{ \begin{array}{l} \begin{array}{cc} y_{u}\leq \mu_{u}-(\sigma_{u}/a) & ,00\\ \mu_{u}-(\sigma_{u}/a)<y_{u}<\mu_{u} & ,01 \end{array}\\ \begin{array}{cc} \mu_{u}\leq y_{u}<\mu_{u}+(\sigma_{u}/a) & ,11\\ y_{u}\geq \mu_{u}+(\sigma_{u}/a) & ,10 \end{array} \end{array}\right.$$

(7) $$K_{u}={\left[Q_{u}(1),Q_{u}(2),\ldots Q_{u}(n)\right]}^{T}$$

There are no wasted channel characteristics so the length of $K_{u}$ is N, where N=2xn. The sequence of $K_{u}$ will be divided into a few blocks $B_{N}$ wherein each block contains 3 bits so $K_{B}=[K_{B}^{T}\ldots K_{B-B_{N}+1}^{T}]$ is obtained. The next step is the synchronization of bit blocks $K_{B}$. The bit blocks will be saved if there are three sequential bits (i.e., 000 or 111) and convert it to 1 or 0. Synchronization is conducted by exchanging wasted index of blocks between the authorized nodes. After synchronization is complete, the remaining block bits will be converted back into bit sequences $Key_{u}$. A detailed description of the SQ method that is run on each node can be seen in Algorithm 1. We initialize 2 parameters i.e., a and k which is able to provide the most optimal configuration as shown in Line 1. Determining the number of areas and bit conversion of each channel characteristic is shown in Line 2–3. The conversion mechanism of channel characteristics measurement results into a multi-bit is shown in Lines 4–14 (Equation (6) and (7)), while the division of bits into several blocks and synchronization of each of the blocks are shown in Lines 15–26. 

**Algorithm 1**: Synchronized Quantization (SQ)

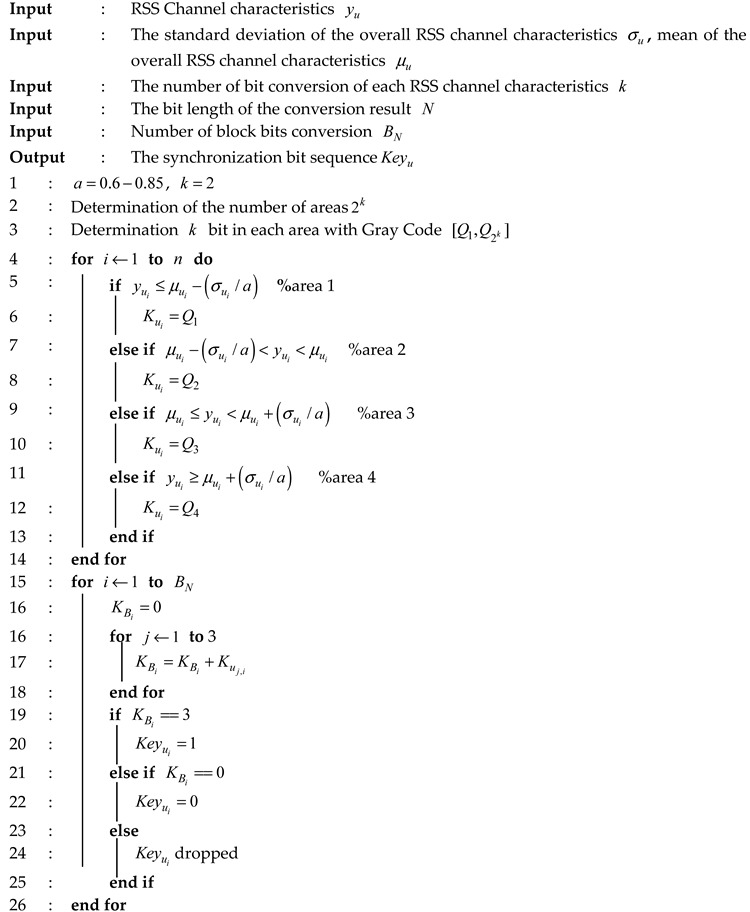



The latter is a privacy amplification phase that consisted of an increased randomness and verification mechanism. Increased randomness mechanism is used to increase the randomization of key synchronization results and remove the possibility of information obtained by the unauthorized node during the block synchronization phase and is used to guess the part of the key [38]. Bit synchronization $Key_{u}$ will be improved for its randomness by using a Universal Hash. The method works by randomly selecting a hash function with certain mathematical properties that can ensure the randomness of produced data. The advantage of this method is the small possibility of obtaining the same data even though the data is selected by the unauthorized node. In this paper, increased randomness was carried out on $Key_{u}$ that had been divided into several blocks of key bits. Each block contains a 128-bit key and will be tested by using NIST software [45]. Blocks that fulfill the requirements will be used as a key to encrypt the plaintext. The verification mechanism is carried out to ensure that the keys used by authorized nodes are the same. In this paper, a block of the 128-bit key that has met the randomness requirements will be hashed using SHA-1. We chose this method because of the high security of the produced hash and it is widely used for one-way functions so key constancy could be seen without revealing information to the tapper. SHA-1 generates hash up to 160 bits long so it will increase the time communications between authorized nodes if all bits are transmitted. Since SHA-1 has the ability to detect bit differences even though it is very small, then only 6 bits of hashes will be sent with a correction capability of up to 98%. The relationship between the length of the bit ℓ and the correction ability c is expressed by Equation (8). 

(8) $$\begin{array}{l} 1-{\left(\frac{1}{2}\right)}^{\ell }\geq c\\ \ell =\left\lceil \log_{1/2}(1-c)\right\rceil \end{array}$$

## 5. Implementation and Performance Evaluation

This section discusses in detail the implementation and performance evaluation of the SSE system. The implementation section provides a detailed description of the devices and software used, as well as the topology measurement scenarios. The performance evaluation section discusses the parameters of performance of the built SSE system, analysis of test results and comparison with several existing systems.

### 5.1. Implementation

**Figure 5:**
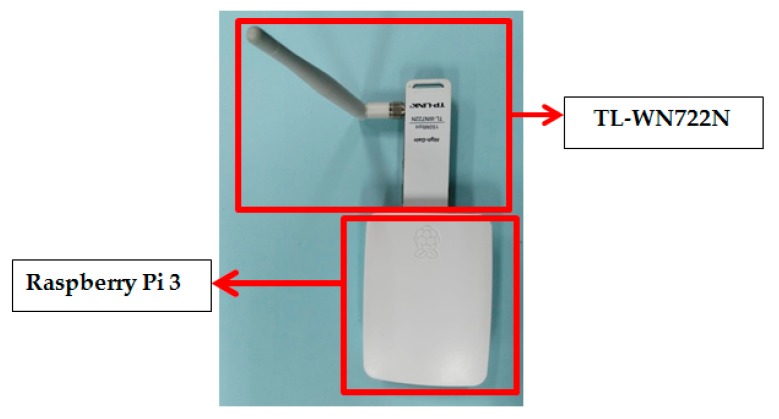
The device used to build the SSE system.

We implemented the SSE system on three Raspberry Pi 3 Model B devices with the operating system Raspbian Stretch and kernel version 4.14.74-v7 +. Two devices become authorized nodes A and B while another becomes an unauthorized node E. One of the reasons why we chose this device is because of the open source operating system used i.e., Linux so there are many possibilities for application development. The number of high-level programming languages that can be used such as Python and the existence of additional slots for various connectivities is also the reason for choosing this device. RSS channel characteristics measurements conducted using the wireless USB Adapter (TL-WN722N) that operates at a carrier frequency of 2.45 GHz. This device works in the half-duplex mode so the measurements must be made alternately. Figure 5 shows the devices that used to build the SSE system. The measurement mechanism is conducted by equipping each node with Wireshark software so that it can capture the received RSS channel characteristics. As an initiator A pings to B at any time interval $t_{m}=110$ ms. The time interval is based on the speed of movement of the authorized nodes which is equal to 1.2 m/s. The Doppler Frequency f obtained is 9.6 GHz (the speed of movement multiplied by the carrier frequency divided by the speed of light) so the coherence time obtained is 104.2 ms (1/|f|). Randomness requirements can be fulfilled if the time interval $t_{m}$ exceeds coherence time [46] so we select time interval 110 ms. In this paper, there are 4000 RSS channel characteristics captured by each node. 

**Figure 6:**
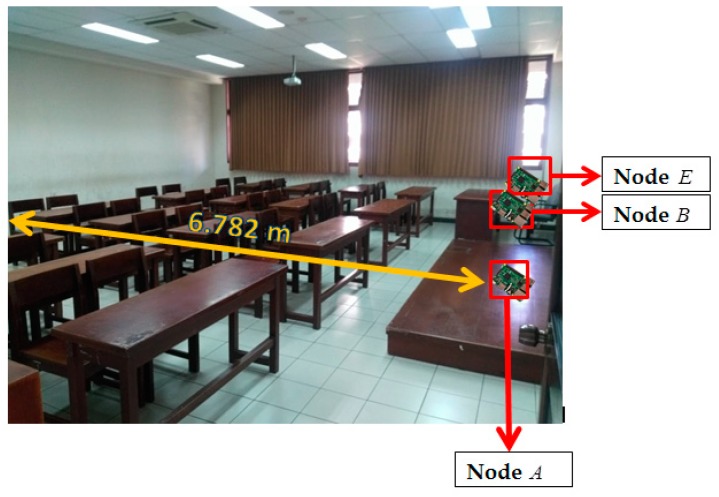
Unobstructed scenario.

**Figure 7:**
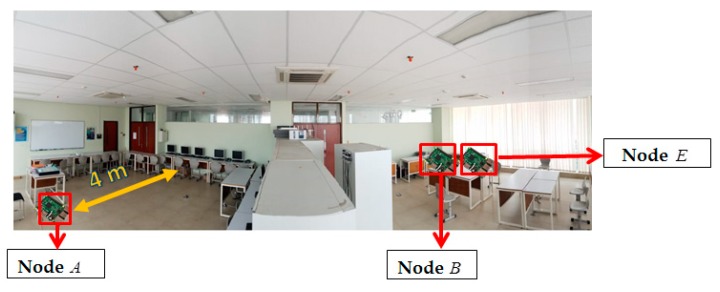
Obstacles scenario.

There are two topology measurement scenarios used in this paper, i.e., unobstructed and obstacles scenarios. In unobstructed scenarios as shown in Figure 6, we measure in classroom measures 8.8 m and 6.9 m. The barrier to the left and right of the classroom is glass, while the front and rear borders are walls. The objects in the classroom include tables, chairs, and blackboards. A is a mobile node and moves straight along the yellow path. B and E are static with a distance of approximately 10 cm. In obstacles scenario, as seen in Figure 7, we measure in two rooms, the laboratory room, and the final project. The room is bounded by closets. Both rooms measure 8 and 14.7 m. The two rooms also contain tables, chairs, blackboards, and closets as a barrier. The same as the previous scenarios A is a mobile node and moves to the yellow path. B and E static with a distance of approximately 10 cm, and separated by closets with A. There were no people passing at the time of measurement in both scenarios.

### 5.2. Performance Evaluation

This section discusses in detail the performance parameters and the performance analysis of test results. The used performance parameters are aimed at determining the success rate of the SSE key generation system. Performance analysis discusses in detail the measurement results performed in two types of scenarios and the advantages of the SSE system compared with previous key generation systems. 

#### 5.2.1. Performance Parameter 

We evaluate the built SSE key generation system by using several parameters i.e., key synchronized rate (KSR), key discrepancy rate (KDR), irregularity, computing time and communication overhead. KSR, computing time and communication overhead are implementational dependent parameters because it is strongly influenced by the computing resources used. KDIR and irregularity are implementational independent parameters because it is influenced by the method used in each key generation phase. So there is no result difference if the key generation system is run in different computing resources. A summary of each parameter is explained as follows.

1. Key synchronized rate (KSR): the number of identical bits during the duration of the key generation system communication. The aim of this proposed key generation system is to conduct key updates every 15 minutes. This time has met the requirements proposed by [47] because the key update time is less than 1 hour.
2. Key discrepancy rate (KDIR): the number of discrepancy bits from the obtained 128-bit key after the quantization phase. The aim of this proposed key generation system is yielding KDIR values of 0 for channel characteristics obtained from authorized nodes and KDIR above 0 for channel characteristics obtained from an unauthorized node.
3. Irregularity: the randomness of the produced key evaluated using 6 tests from the National Institute of Standards and Technology (NIST) [45]. The tests included frequency test (F), frequency block test (BF), runs test (R), a long run of ones in the block (LROB), approximate entropy test (AP), and cumulative sums test (CS). Each test result will produce P value. If P value is at least 0.01 then the produced key has met the randomness requirements.
4. Computing time: the length of time required to complete each key generation phase. The lower the complexity of the phases performed, the faster the resulted in computing time is, so it is suitable for IoT devices that have limited computing resources.
5. Communication overhead: the number of bytes transmitted for two-node communication. In the SSE system, communication overhead is greatly influenced by the number of wasted index of blocks in the quantization phase and the number of hashes transmitted during privacy amplification.

#### 5.2.2. Performance Analysis of the SSE System

**Figure 8:**
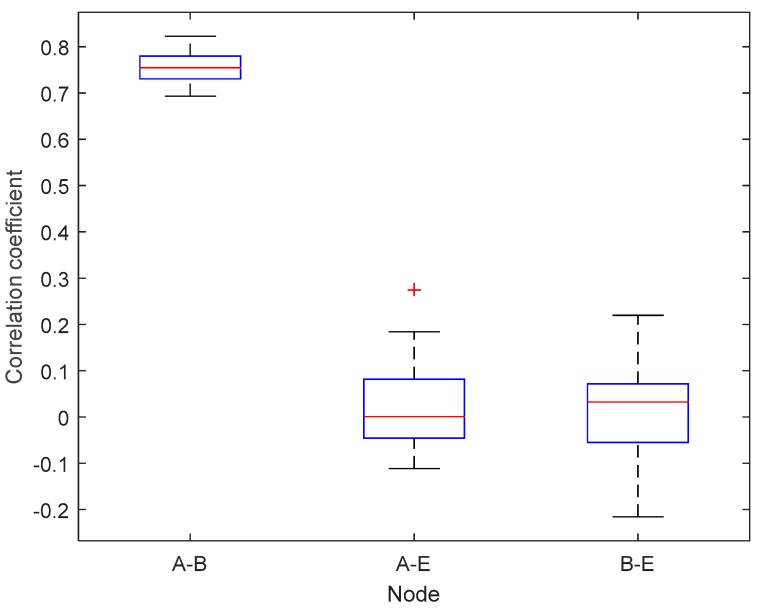
Box plots of the correlation coefficient value in the unobstructed scenario.

**Figure 9:**
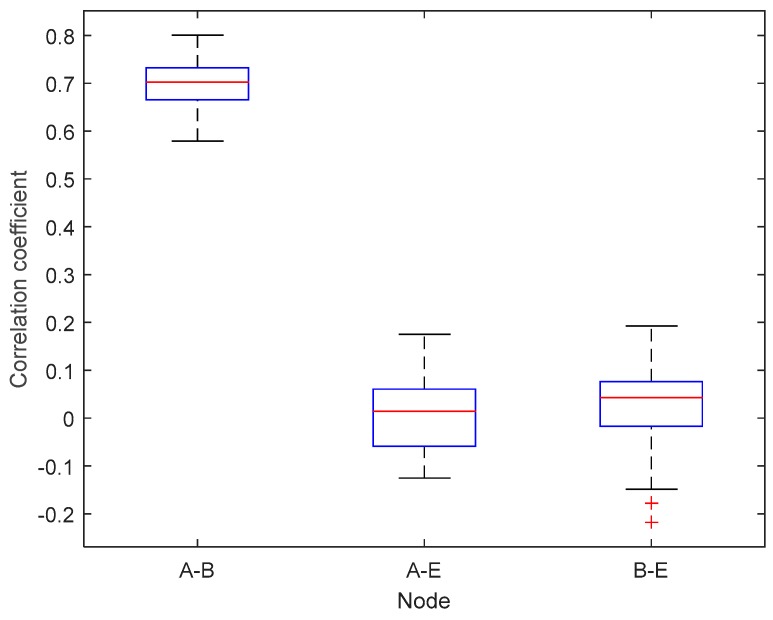
Box plots of the correlation coefficient value in the obstacles scenario.

Figure 8 and Figure 9 show box plots of the correlation coefficients measurement results between nodes in the unobstructed and obstacles scenarios. In this paper, the box plots were obtained from the calculation of data block correlation coefficients with Equation (1), each of which contained 128 RSS channel characteristics. The smaller the value of the correlation coefficient, the more different RSS channel characteristic values are obtained from the measurement results, so it is difficult to get the same keys. The correlation coefficients value range from 1 to −1. There are three pieces of information that will be retrieved from the box plots i.e., the median, the lower and upper quartile of the correlation coefficient. The correlation coefficient results between nodes A−B in the unobstructed scenario obtained a median value of 0.7547, lower quartile of 0.7303, and the upper quartile of 0.7809. The correlation coefficient results between nodes A−E show a median value of 0.0007, lower quartile of −0.0462 and upper quartile of 0.0825. Meanwhile, the correlation coefficient obtained between nodes B−E indicates similar results with correlation coefficient obtained from the node A−E with the median value of 0.0323, the lower quartile and the upper quartile of −0.0575 at 0.0718. The results of testing in an unobstructed scenario indicate the difficulty of the unauthorized node to get the same key because of the significant difference of the correlation coefficient between the authorized nodes (A−B) and the unauthorized node (A−E and B−E). The correlation coefficient results between nodes A−B in the obstacles scenario obtained a median value of 0.7021, lower quartile of 0.6645, and the upper quartile of 0.7326. The correlation coefficient results between nodes A−E show a median value of 0.0143, lower quartile of −0.0687 and the upper quartile of 0.0631. Whereas the correlation coefficient obtained between nodes B−E indicate similar results with correlation coefficient obtained from the node A−E with the median value of 0.0430, lower quartile of −0.0220 and upper quartile of 0.0772. Generally, it can be seen that the correlation coefficient value of authorized nodes in the obstacles scenario is lower than the unobstructed scenario. There is a barrier between authorized nodes so that the RSS channel characteristics obtained is also worse and decreases the correlation coefficient of the measurement results. Similar to the testing conducted in an unobstructed scenario, the correlation coefficients obtained also indicate the difficulty of the unauthorized node to get the same key because of the significant difference of the correlation coefficient between the authorized nodes (A−B) and the unauthorized node (A−E and B−E).

**Figure 10:**
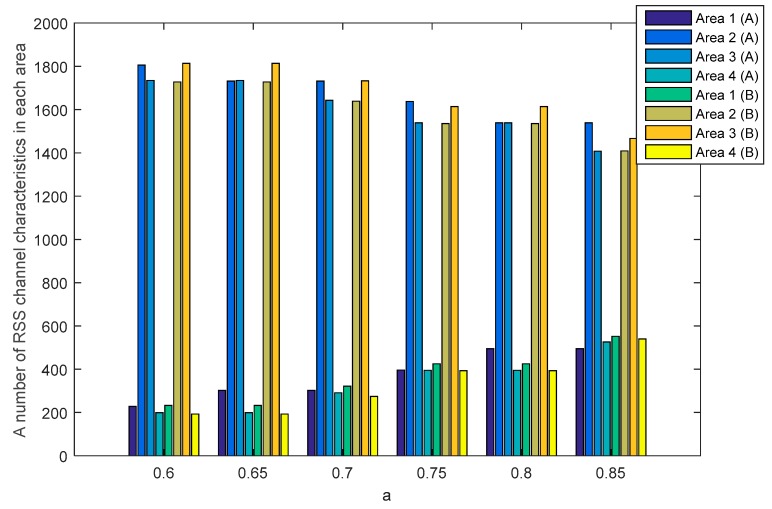
A number of RSS channel characteristics (node A−B) in each area in the unobstructed scenario.

**Figure 11:**
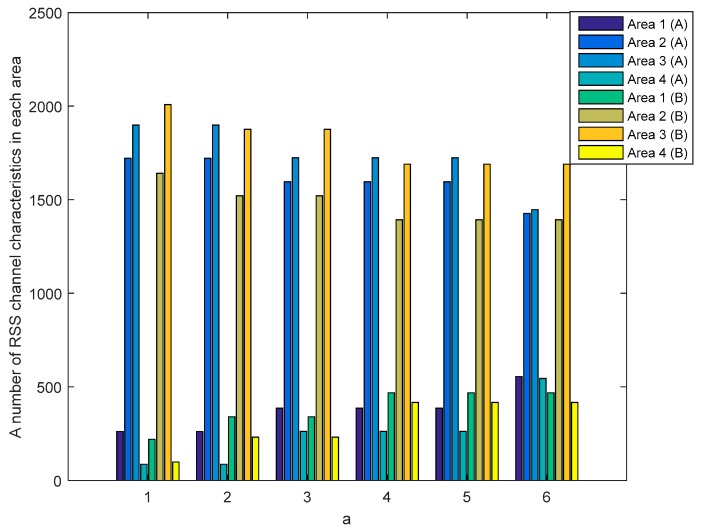
A number of RSS channel characteristics (node A−B) in each area in the obstacles scenario.

The SQ method performs the multi-bit conversion by dividing the RSS channel characteristics into several areas. The number of RSS channel characteristics in each area is strongly influenced by the selection of a as a standard deviation dividing parameter. Figure 10 and Figure 11 show the number of RSS channel characteristics from the node A and B in each area on the unobstructed and obstacles scenarios. The performed tests indicate that in all of the value of a the highest amount of RSS is in area 2 and 3. These conditions resulted in increasing the probability of getting three sequential bits i.e., 111 and convert it to 1. The resulting key bit will be dominated by 1. The higher the a value, the higher the possibility to get variations 1 and 0 of the produced keys is. This happens because of the growing number of RSS channel characteristics that are in area 1 and 4 thus increasing the probability of getting three sequential bits i.e., 000 and converting them to 0. The higher variations 1 and 0 in the resulting key, the higher the likelihood of different key bits being produced between the two nodes. Table 2 shows the number of equal keys with lengths of 128, 192 and 256 that were successfully produced from two scenarios. Equal keys are obtained if the resulting KDIR between authorized nodes is 0. The test results in unobstructed scenario show that the higher the value of a the less equal key is successfully produced. This happens because of the higher variations of the 1 and 0 thus reducing the possibility of getting an equal key. The highest number of equal keys is obtained when a=0.6 and a=0.65. In these parameters, most RSS channel characteristics are in areas 2 and 3 so that the resulting key bits are dominated by 1. The low variation in the resulting bits increases the possibility to get an equal key. The test results in obstacles scenario show that the equal key is only obtained when a=0.6 and a=0.65. The high difference in the amount of RSS channel characteristics in each area and also the higher variations of 1 and 0 triggering more difficulties to get the equal key. The encryption method used to randomize the messages is Advanced Encryption Standard (AES) with key lengths of 128, 192, and 256. In this study, we focus on 128-bit keys because our proposed key generation system produces more keys at the key length of 128. The more keys produced, the higher the KSR value is generated so it can improve the performance of the built key generation system. 

**Figure 12:**
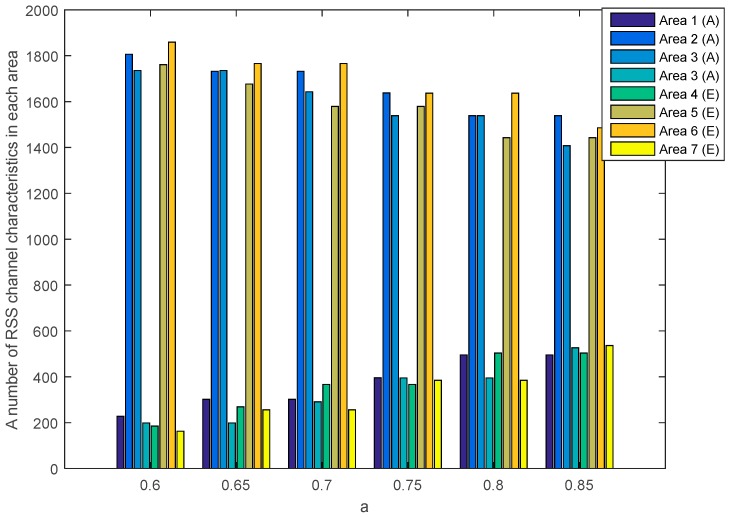
A number of RSS channel characteristics (node A−E) in each area in the unobstructed scenario.

**Figure 13:**
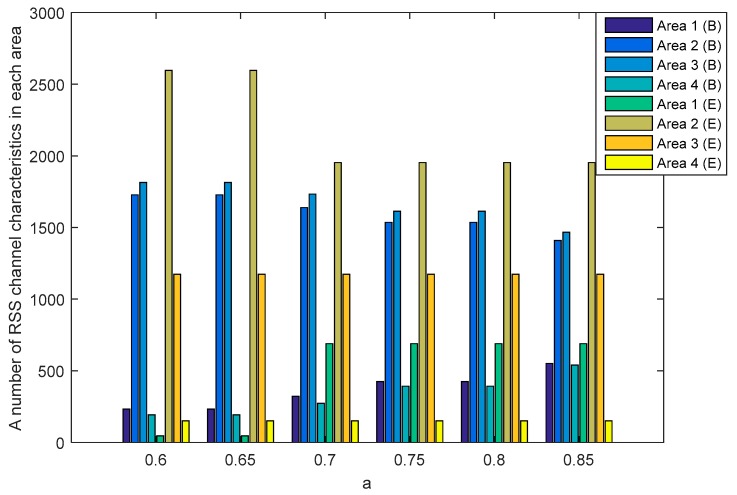
A number of RSS channel characteristics (node B−E) in each area in the unobstructed scenario.

**Figure 14:**
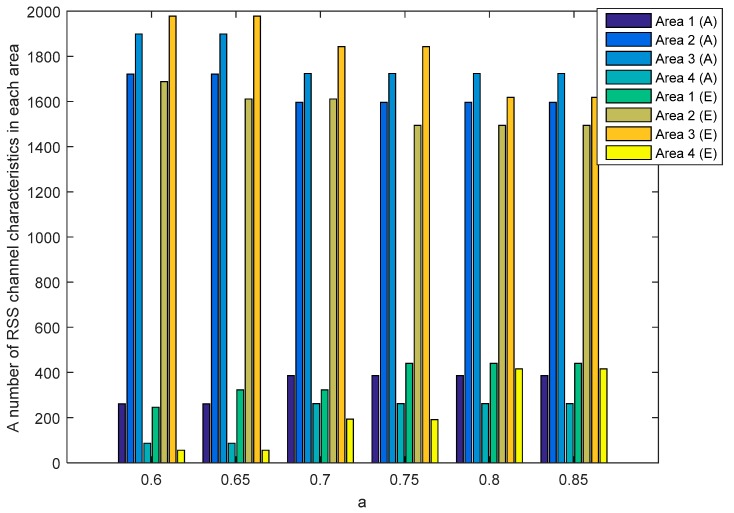
A number of RSS channel characteristics (node A−E) in each area in the obstacles scenario.

**Figure 15:**
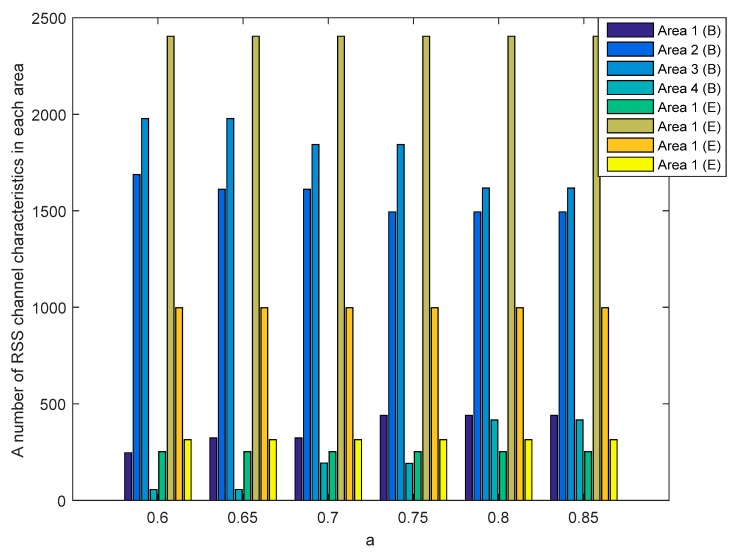
A number of RSS channel characteristics (node B−E) in each area in the obstacles scenario.

Figure 12 and Figure 13 show the number of RSS channel characteristics from nodes A and E (node A−E) and nodes B and E (node B−E) in each area in the unobstructed scenario. Meanwhile, Figure 14 and Figure 15 show the number of RSS channel characteristics from nodes A and E (node A−E) and nodes B and E (node B−E) in each area in the obstacles scenario. At the node A−E, RSS channel characteristics that are processed by the node E is RSS channel characteristics received from the node A. At the node B−E, RSS channel characteristics that are processed by the node E is RSS received from the node B. From all scenarios, it can be seen that the number of RSS channel characteristics from a node A−E is mostly in areas 2 and 3 for all values of a. These conditions result in the higher probability of getting three sequential bits i.e., 111 and convert it to 1. However, testing in obstacles scenario results in the possibility of obtaining three sequential bits, i.e., 111 which are higher when compared to unobstructed scenarios because there are more numbers of RSS channel characteristics in areas 2 and 3. These results in the higher keys are produced. The number of RSS channel characteristics from a node B−E is also mostly in areas 2 and 3 for all values a, but there are significant differences in the number of RSS channel characteristics from area 2 and 3. These results in the lower probability of getting three sequential bits i.e., 111 so fewer keys are produced.

The number of produced 128-bit keys by the unauthorized node and key discrepancy rate (KDIR) between the unauthorized and authorized nodes is shown in Table 3, Table 4, Table 5, Table 6 and Table 7. The produced 128-bit keys by the authorized nodes are expressed as Ka (node A−B) while the produced 128-bit keys by the unauthorized node are expressed as Ke (node A−E) and Kb (node B−E). The KDIR value was obtained from the number of discrepancy bits between Ka and Ke with Ka and Kb. To meet security requirements, it is expected that there is no equal 128-bit key obtained by the unauthorized node indicated by the KDIR value above 0. The performed test results in both scenarios indicate less number of produced 128-bit keys by node B−E compared to the node A−E. This happens because of the low probability of getting three sequential bits i.e., 111 because of the significant difference in the number of RSS in areas 2 and 3. The fewer three sequential bits produced, the fewer 128-bit keys that can be produced. The test results in unobstructed scenarios also indicate that the higher the value of a, the higher KDIR value between unauthorized and authorized nodes. The high KDIR is caused by the higher variations of the 1 and 0 so as to increase the difference in the produced bits. From the results of testing in the obstacles scenario, there is no significant difference in the KDIR value between the values of a=0.6 and a=0.65. This happens because the number of RSS is almost the same in each area for both a values. Overall, it can be said that the built SSE system has met the security requirements because all KDIR values have exceeded 0. There are no equal keys that were successfully produced by the unauthorized node. 

The computation of time testing is conducted by calculating the time needed in the SQ and privacy amplification phase. In the SQ phase, there are two calculated times, i.e., computing time (CT) of multi-bit conversion and the division of bits into several blocks, as well as communication/synchronization time (C/ST) of exchanges of wasted index of blocks to ensure the produced key is absolutely the same as it can eliminate the error-correcting phase. In the privacy amplification phase, there are also two calculated times, i.e., computing time (CT) of increased randomness and the verification mechanism, as well as communication/synchronization time (C/ST) of hash exchanges between the two authorized nodes. Communication overhead testing is conducted by calculating the size of the files sent for synchronization between 2 authorized nodes. Synchronization is conducted after the SQ and the privacy amplification phase. Table 8 and Table 9 show the computing time testing to get equal 128-bit keys for each a value in two scenarios while communication overhead testing is shown in Table 10 and Table 11. The results of the tests showed no significant computing time differences for each a value in both scenarios, even in the SQ or privacy amplification phases. Computing time differences actually occur between SQ and privacy amplification phases. This happens because the processed data in the SQ phase for each a value is the same channel characteristics measurement data. The processed data in the privacy amplification phase is the remaining of the SQ phase data. Communication/synchronization time testing also showed no significant time difference for each a value in both the SQ and privacy amplification phases. This happens because there is no significant difference between the produced communication overhead. 

**Figure 16:**
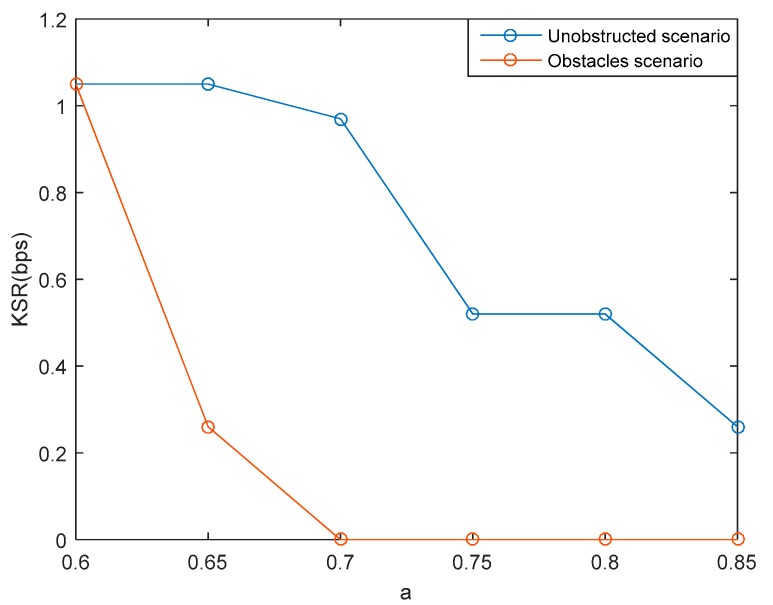
Key synchronized rate (KSR) for several variations of a value.

In this paper, the KSR parameter is obtained by calculating the number of equal key bits during the duration of the SSE system communication. The duration of this system communication includes the measurement of channel characteristics, computing and communication/synchronization time at the synchronized quantization (SQ) phase, as well as computing and communication/synchronization time at the privacy amplification phase. This study carried out testing by varying the parameter of a within 0.6 to 0.85. The varied selection of the value of a is based on the attempt to get the equal 128-bit keys between authorized nodes and ensuring that there are no equal 128-bit keys of the unauthorized node. Figure 16 shows that the higher the value of a, the lower the generated KSR as there are fewer number of equal 128-bit keys. This happens because there is an increase of 1 and 0 of the produced key bits, thus minimizing the possibility of obtaining an equal key. The results of the performed tests in unobstructed scenarios showed that the highest KSR was obtained at a=0.6 and a=0.65, i.e., 1.05 bps, while the lowest KSR was obtained at a=0.85 i.e., 0.26 bps. The obtained values indicate that the computing time to get 128-bit keys ranges from 2.03 min to 8.21 min. Meanwhile, the results of the testing carried out in unobstructed scenarios showed that the highest KSR was obtained at a=0.6, i.e., 1.05 bps, while the lowest KSR was obtained at a=0.65, i.e., 0.26 bps. The obtained values indicate that the computing time to get 128-bit keys ranges from 2.03 min to 8.21 min. Overall, it can be said that the SSE system is able to produce keys below 1 hour to meet the requirements of the update key maximum time proposed by [44]. 

Irregularity testing was carried out to ensure that the 128-bit keys of universal hash results met the randomness requirements. In this paper, the authors used the 128-bit keys produced by a=0.6. This value is selected because of the higher number of the produced 128-bit keys than the other value in both scenarios. In addition, the security requirements have also been met because there are no equal 128-bit keys produced by the unauthorized nodes. There were 6 randomization tests performed, i.e., frequency test (F), frequency block test (BF), runs test (R), a long run of ones in the block (LROB), approximate entropy test (AP), and cumulative sums test (CS). Each test result will produce a P value which is the randomness probability value of 128-bit keys (0≤P value ≤1). The closer to 1, the more random the 128-bit keys are produced. In order to fulfill the randomization requirements, the minimum P value of the 128-bit keys is 0.01. The details of the usability of each test are explained as follows. The F test is used to determine the ratio of 0 and 1 of 128 key bits. The BF test aims to discover the ratio of 1 of each block in the 128-bit keys. The determination of whether fluctuations 0 or 1 in a 128-bit key is too fast or slow can be known by R tests, while the determination of whether fluctuations of 0 or 1 of each block in a 128-bit key is too fast or slow can be known with the LROB test. The AP test is used to determine the frequency of all possible intersection bits of each block in the 128-bit keys. Meanwhile, the CS test is used to specify that the accumulative number of the 128 key bits tested is too large or too small for the accumulative sum of the random sequence. Table 12 shows the results of the NIST test in the unobstructed and obstacles scenario. In both scenarios, it appears that all the produced keys have met the randomness requirements for 6 tests because the resulting P value has exceeded the specified standard, i.e., 0.01. The ranking of keys to be selected on the SSE system is Ka-4, Ka-1, Ka-2, and Ka-3. The selection of this ranking is based on the results of the AP test. If Ka-4 fails to be used as a key in the verification phase, then Ka-1 will be used as such a key. The highest P value of the AP tests is 0.9484 (unobstructed scenarios) and 0.9403 (obstacles scenario). This shows that K-4 obtained during the obstacles scenario has a higher persistence bit compared to K-4 obtained when in the unobstructed scenario.

#### 5.2.3. Comparison of Performance between SSE Systems and Existing Key Generation Systems

There are two performance parameters, i.e., computing time and the communication overhead, that are used to compare SSE systems with previous key generation systems that are also implemented on IoT devices with limited power and computing. The selection of these parameters is based on the consideration of obtaining an efficient key generation system in terms of computing time and the communication overhead. The lower the computing time and the communication overhead required, the more efficient the built key generation system. There are 4 existing key generation systems that will be used for comparison, i.e., system [9,31,33,46,48]. The system [9] uses four phases where the RSS channel characteristics will be quantized using the cumulative distribution function (CDF) method. The system [31] uses five phases where the RSS channel characteristics will be divided into several blocks, each containing 50 data. Each block will be pre-processed using the Kalman method and the results will be converted into multi-bits with the quantization method proposed by [24]. The system [33] uses five phases in building a key generation system. The RSS channel characteristics will be pre-processed using a third order polynomial regression. The results of the signal pre-process phase will be divided into several blocks, each containing 250 bits. Each block will be converted into multi-bit with the quantization method proposed by [24]. The system [47] works by signal pre-processing the RSS channel characteristics using the discrete cosine transform (DCT) method. The results of the pre-process will be quantized using several parameters which include the mean and standard deviation. The system [48] works using 4 phases where the RSS channel characteristics will be divided into several blocks, each containing 128 bits. Each block will be converted into multi-bit with the quantization method proposed by [38]. All of the existing key generation systems still require an error-correcting phase to correct the different bits produced by the authorized nodes. In this paper, the error-correcting method used is BCH (255, 87), while privacy amplification uses the Universal Hash and SHA-1 methods. 

Computing time system testing in both scenarios is divided into two parts, i.e., parts A and B. In the SSE system, part A consists of the SQ phase while part B consists of the privacy amplification phase. In the existing key generation system, part A consists of a signal pre-processing phase to the error-correcting phase, while part B consists of privacy amplification. Each part will be divided into two, i.e., computing time (CT) and communication/synchronization time (C/ST). Table 13 shows a comparison of computing time between the SSE and the existing system. The results of the performed tests indicate that the SSE system is able to reduce computing time (CT) of part A to 25.77 times (unobstructed scenarios) and 26.08 times (obstacles scenario) compared to existing systems. The decrease in communication/synchronization time (C/ST) reaches 1.55 times (unobstructed scenarios) and 1.52 times (obstacles scenario). The test results of part B also show that SSE systems are able to reduce computing time (CT) to 2.60 times (unobstructed scenario) and 2.47 times (obstacles scenario) compared to existing systems. There is no significant communication/synchronization (C/ST) time difference of part B between SSE and the existing systems because the produced communication overhead is almost the same. Overall, it can be seen that the computing time (CT) and communication/synchronization (C/ST) time of the SSE system are lower than the existing system. This happens because, in part A, the multi-bit conversion of the SSE system is conducted directly from the RSS channel characteristics without going through the signal pre-processing phase. Further, there is no blocking division in processing RSS channel characteristics so it speeds up computing time compared to existing systems. The high computing time of existing systems is due to the error-correcting phase. The more data proceeded at this phase, the higher the computing time, communication/synchronization time and communication overhead required. Table 14 shows a comparison of the communication overhead between the SSE system and existing systems. The communication overhead is calculated from the size of the file sent for synchronizing the authorized nodes. In the SSE system, the data synchronization occurs after the SQ and privacy amplification phase, whereas in existing systems, the data synchronization occurs after the error-correcting and privacy amplification phase. The results of the performed tests indicate that the SSE systems can reduce the communication overhead from part A to 2.75 times (unobstructed scenario) and 2.92 times (obstacles scenario) compared to existing systems. There is no significant communication overhead difference of part B between SSE and the existing systems because of produced the communication overhead is almost the same. This happens because synchronization after the SQ phase is only conducted on the index of the wasted data block, while synchronization after the error-correcting phase is carried out on the parity bit to increase the size of the communication overhead. Overall, the results of the tests conducted indicate that SSE systems are able to reduce computing time, communication/synchronization time and the communication overhead compared to existing systems. Therefore, it can be said that the SSE system is more efficient for implementation of IoT devices with limited resources compared to existing systems. 

## 6. Conclusions

This paper proposes the SSE system as an efficient key generation system and synchronized quantization (SQ) as a part of the SSE system that synchronizes data blocks in the quantization phase in order to eliminate the signal pre-processing and error-correcting phase. The efficiency of the system is indicated by lower computing time and the communication overhead between authorized nodes compared to existing systems. Validation of the SSE system performance is carried out in two real indoor environment scenarios, i.e., unobstructed and the obstacles scenarios. The test results showed that the SQ method was able to produce equal 128-bit keys without going through the signal pre-processing and error-correcting phase with KSR values reaching 1.05 bps (unobstructed and obstacles scenario). Testing of computing time and the communication overhead in two scenarios also show the efficiency of the SSE system compared to the existing system. This is indicated by a decrease in computing time up to 25.77 times (unobstructed scenarios) and 26.08 times (obstacles scenario), and decreased communication/synchronization time up to 1.55 times (unobstructed scenarios) and 1.52 times (obstacles scenario) of part A. Part B also shows that SSE systems are able to reduce computing time to 2.60 times (unobstructed scenario) and 2.47 times (obstacles scenario). The communication overhead of part A also decreased by 2.75 times (unobstructed scenario) and 2.92 times (obstacles scenario). In addition, the built SSE system has met the security requirements because there is no equal key that was successfully produced by the unauthorized node. 

## Figures and Tables

**Table 1:** Comparison of the key generation phases.

Key Generation System	Key Generation Phases				
	Measuring Channel Characteristics	Signal Pre-Processing	Quantization	Error Correcting	Privacy Amplification
[22,23,24,25,26,27]	V	V	V	V	V
[30]	V	V	V	-	V
[38,39,40]	V	-	V	V	V
SSE System	V	-	V	-	V

**Table 2:** A number of equal keys (node A−B).

Number of Bit Keys	a	Number of Equal Keys	
		Unobstructed Scenario	Obstacles Scenario
128	0.6	4	4
	0.65	4	1
	0.7	3	-
	0.75	2	-
	0.8	2	-
	0.85	1	-
192	0.6	2	3
	0.65	2	-
	0.7	2	-
	0.75	1	-
	0.8	1	-
	0.85	-	-
256	0.6	2	2
	0.65	2	-
	0.7	1	-
	0.75	1	-
	0.8	1	-
	0.85	-	-

**Table 3:** A number of keys (node A−E and B−E).

Scenarios	Number of Keys Ke for Each a Value						Number of Keys Kb for Each a Value					
	0.6	0.65	0.7	0.75	0.8	0.85	0.6	0.65	0.7	0.75	0.8	0.85
Unobstructed scenario	2	2	2	2	1	1	1	1	1	1	1	1
Obstacles scenario	3	1	-	-	-	-	3	1	-	-	-	-

**Table 4:** The key discrepancy rate of unauthorized nodes in the unobstructed scenario (a=0.6 and a=0.65).

KDIR for Each a Value									
a	Ka	Ke-1	Ke-2	Kb-1	a	Ka	Ke-1	Ke-2	Kb-1
0.6	Ka-1	0.0391	0.0469	0.0234	0.65	Ka-1	0.0547	0.0703	0.0234
	Ka-2	0.0703	0.0781	0.0547		Ka-2	0.0938	0.1094	0.0625
	Ka-3	0.0703	0.0781	0.0547		Ka-3	0.0703	0.1016	0.0547
	Ka- 4	0.0703	0.0781	0.0547		Ka- 4	0.0859	0.1016	0.0703

**Table 5:** The key discrepancy rate of unauthorized nodes in the unobstructed scenario (a=0.7 and a=0.75).

KDIR for Each a Value								
a	Ka	Ke-1	Ke-2	Kb-1	a	Ka	Ke-1	Kb-1
0.7	Ka-1	0.1172	0.1172	0.2891	0.75	Ka-1	0.1406	0.2969
	Ka-2	0.1406	0.1406	0.3125		Ka-2	0.1875	0.2969
	Ka-3	0.1484	0.1328	0.2734		-	-	-

**Table 6:** The key discrepancy rate of unauthorized nodes in the unobstructed scenario (a=0.8 and a=0.85).

KDIR for Each a Value							
a	Ka	Ke-1	Kb-1	a	Ka	Ke-1	Kb-1
0.8	Ka-1	0.2109	0.3047	0.85	Ka-1	0.2969	0.4063
	Ka-2	0.2344	0.3281		-	-	-

**Table 7:** The key discrepancy rate of unauthorized nodes in the obstacles scenario (a=0.6 and a=0.65).

KDIR for Each a Value											
a	Ka	Ke-1	Ke-2	Ke-3	Kb-1	a	Ka	Ke-1	Ke-2	Ke-3	Kb-1
0.6	Ka-1	0.0625	0.0703	0.0547	0.0547	0.65	Ka-1	0.0547	0.0781	0.0547	0.0391
	Ka-2	0.0391	0.0469	0.0313	0.0313		-	-	-	-	-
	Ka-3	0.0547	0.0625	0.0469	0.0469		-	-	-	-	-
	Ka-4	0.0469	0.0547	0.0391	0.0391		-	-	-	-	-

**Table 8:** Testing of computing time for each a value in the SQ phase.

Scenarios	Computing Time (CT) (s)						Communication/Synchronization Time (C/ST) (s)					
	0.6	0.65	0.7	0.75	0.8	0.85	0.6	0.65	0.7	0.75	0.8	0.85
Unobstructed scenario	24.54	24.35	24.41	24.45	24.42	24.51	4.23	4.33	4.41	4.40	4.39	4.48
Obstacles scenario	24.30	24.32	-	-	-	-	4.32	4.33	-	-	-	-

**Table 9:** Testing of computing time for each a value in the privacy amplification phase.

Scenarios	Computing Time (CT) (s)						Communication/Synchronization Time (C/ST) (s)					
	0.6	0.65	0.7	0.75	0.8	0.85	0.6	0.65	0.7	0.75	0.8	0.85
Unobstructed scenario	15.05	15.19	15.28	15.12	15.23	15.17	4.22	4.25	4.31	4.27	4.33	4.35
Obstacles scenario	15.42	15.42	-	-	-	-	4.23	4.24	-	-	-	-

**Table 10:** Testing of communication overhead for each a value in the SQ phase.

Scenarios	Communication Overhead (byte)					
	0.6	0.65	0.7	0.75	0.8	0.85
Unobstructed scenario	17962	17904	18381	18852	18791	19460
Obstacles scenario	16995	17361	-	-	-	-

**Table 11:** Testing of communication overhead for each a value in the privacy amplification phase.

Scenarios	Communication Overhead (byte)					
	0.6	0.65	0.7	0.75	0.8	0.85
Unobstructed scenario	11276	11276	11270	11270	11270	11270
Obstacles scenario	11276	11276	-	-	-	-

**Table 12:** NIST Test.

Tes	P Value of Unobstructed Scenario				P Value of Obstacles Scenario			
	Ka-1	Ka-2	Ka-3	Ka-4	Ka-1	Ka-2	Ka-3	Ka-4
	(A-B)	(A-B)	(A-B)	(A-B)	(A-B)	(A-B)	(A-B)	(A-B)
F	0.4795	1.0000	0.2159	1.0000	0.3768	0.8597	1.0000	1.0000
BF	0.1532	0.0239	0.3540	0.2202	0.9170	0.9326	0.4530	0.8666
R	0.8941	0.5959	0.2260	0.4795	0.1356	0.2900	0.5959	0.4795
LROB	0.4098	0.9404	0.7529	0.4203	0.1806	0.0359	0.8893	0.5788
AP	0.4540	0.3491	0.0863	0.9484	0.2941	0.4552	0.2806	0.9403
CS (fwd)	0.5748	0.6547	0.3697	0.7375	0.6548	0.8188	0.9842	0.3697
CS (rvs)	0.1542	0.6547	0.3697	0.7375	0.6548	0.9493	0.9842	0.3697

**Table 13:** Comparison of computing time between the SSE and the existing system.

Key Generation System	Unobstructed Scenario				Obstacles Scenario			
	Part A		Part B (s)		Part A (s)		Part B (s)	
	CT(s)	C/ST(s)	CT(s)	C/ST(s)	CT(s)	C/ST(s)	CT(s)	C/ST(s)
SSE system	24.54	4.23	15.05	4.22	24.30	4.32	15.42	4.23
System [9]	506.03	6.44	28.60	4.23	509.31	6.55	31.17	4.32
System [31]	622.63	6.50	31.79	4.51	623.25	6.45	29.78	4.30
System [33]	496.59	6.35	27.49	4.33	490.38	6.44	24.79	4.47
System [46]	632.43	6.56	39.20	4.97	633.82	6.45	38.12	4.55
System [48]	500.95	6.32	25.33	4.43	498.18	6.44	26.14	4.33

**Table 14:** Comparison of communication overhead between the SSE and the existing system.

Key Generation System	Unobstructed Scenario		Obstacles Scenario (Byte)	
	Communication Overhead of Part A (Byte)	Communication Overhead of Part B (Byte)	Communication Overhead of Part A (Byte)	Communication Overhead of Part B (Byte)
SSE system	17,962	11,276	16,995	11,276
System [9]	49,339	11,403	50,133	11,908
System [31]	49,431	11,276	49,608	11,276
System [33]	47,289	11,546	47,033	12,100
System [46]	49,127	11,932	48,582	11,624
System [48]	48,513	12,005	48,680	11,707
